# Impact of anti-VEGF therapy on distinctive retina layers in patients with macular edema secondary to branch retinal vein occlusion

**DOI:** 10.1186/s12886-023-02981-7

**Published:** 2023-05-25

**Authors:** Hui Wang, Chanjuan Wang, Shaochi Zhang, Jun Liu, Xiaojun Bi

**Affiliations:** 1 Ophthalmology Center of Changzhi People’s Hospital affiliated to Changzhi Medical College, Changzhi City, China; 2 Ningxia Eye Hospital, People Hospital of Ningxia Hui Autonomous Region (First Affiliated Hospital of Northwest University for Nationalities), Yinchuan, 750000 China

**Keywords:** Anti-vascular endothelial growth factor, Branch retinal vein occlusion, Macular edema, Retinal layer segmentation, Optical coherence tomography

## Abstract

**Background:**

To explore the impact of anti-vascular epithelial growth factor (ant-VEGF) on the thickness of each retinal layer in patients with macular edema (ME) secondary to the branch retinal vein occlusion (BRVO).

**Methods:**

This retrospective study included patients with ME secondary to monocular BRVO who received anti-VEGF therapy in Ningxia Eye Hospital between January-December 2020.

**Results:**

Forty-three patients (25 males) were included, with 31 showed > 25% reduction in central retinal thickness (CRT) after anti-VEGF therapy (response group), and the others showed a ≤25% reduction in CRT (no-response group). The response group showed significantly smaller mean changes in the ganglion cell layer (GCL) (after 2 months) and inner plexiform layer (IPL) (after 1, 2, and 3 months) and significantly greater mean changes in the inner nuclear layer (INL) (after 2 and 3 months), outer plexiform layer (OPL) (after 3 months), outer nuclear layer (ONL) (after 2 and 3 months), and CRT (after 1 and 2 months) (all P < 0.05) as compared to the no-response group. The mean change in the thickness of each retinal layer IPL (P = 0.006) between the two groups was significantly different after controlling for a time and with a significant time trend (P < 0.001). Additionally, patients in the response group were more likely to have an improvement in IPL (43.68 ± 6.01 at 1 month and 41.52 ± 5.45 at 2 months vs. 39.9 ± 6.86 at baseline) after anti-VEGF therapy, while those in no response group might show improvement in GCL (45.75 ± 8.24 at 1 month, 40.00 ± 8.92 at 2 months, and 38.83 ± 9.93 at 3 months vs. 49.67 ± 6.83 at baseline).

**Conclusions:**

Anti-VEGF therapy might help restore the retinal structure and function in patients with ME secondary to BRVO, and those who have a response after anti-VEGF therapy are more likely to improve IPL, while those having no response might show improvement in GCL.

## Background

Retinal vein occlusion (RVO) is the partial or complete obstruction of a retinal vein that commonly occurs in adults after 40 years and with a history of hypertension, hyperlipidemia, or diabetes. Its worldwide prevalence was 0.5% in adults in 2008 [1–3]. Diabetes is an important risk factor for RVO [4, 5]. Partial or complete RVO reduces the venous return from the retinal circulation and results in vascular leakage within the retinal circulation, which may lead to intraretinal hemorrhages, retinal ischemia, increased intravitreal pressure, macular edema (ME), and ultimately vision loss [1–3]. RVO is divided into branch RVO (BRVO) and central RVO (CRVO). About 30% of patients with BRVO develop ME [1–3], which destroys the retina’s morphological structure and significantly impacts vision [6].

Two major complications are associated with BRVO: ME and retinal ischemia. ME is an important cause of vision loss and visual impairment, whereas retinal ischemia causes iris and retinal neovascularization. It is believed that BRVO causing ME increases the capillary pressure caused by the retinal vein thrombosis, resulting in increased capillary permeability and leakage of tissue fluid and blood to the retina [1]. The co-occurrence of retinal ischemia and hypoxia can generate vascular endothelial growth factor (VEGF), which in turn can potentially enhance the capillary permeability and promote tissue fluid and blood leakage into the extracellular space, thus leading to the further development of ME [2]. The VEGF receptor-2 (VEGFR-2) causes the phosphorylation of closure protein, atretic zone-1, and vascular endothelial cadherin, destroying tight connections. It also promotes mitosis and neovascularization, resulting in increased capillary permeability and accumulation of fluid inside or outside the cell and the outer layer of the retina. These findings indicate that VEGF could play a vital role in the pathogenesis of BRVO-CME. Recently, anti-VEGF therapy has been used as the primary treatment for BRVO-CME [3, 6–8].

VEGF plays a vital role in the occurrence of ME secondary to RVO [3, 7]. The main anti-VEGF drugs include ranibizumab, aflibercept, and conbercept [8]; conbercept and ranibizumab are considered safe and effective in treating RVO, as indicated by no significant differences in their long-term efficacy [8]. Therefore, intravitreal anti-VEGF drugs are becoming the compelling choice for treating RVO [1, 2], and their injection into patients with RVO significantly improves ME [8].

The morphological changes in the inner plexiform layer (IPL), inner nuclear layer (INL), outer plexiform layer (OPL), outer nuclear layer (ONL), and photoreceptor layer (PL) of the retina secondary to BRVO are related to prognosis [9, 10]. Nevertheless, a large amount of clinical data suggests that several patients with BRVO show different morphological changes of ME, edema in different parts of the retinal interlayer capsule, and different degrees of damage to each layer of the retina after anti-VEGF treatment [11–13]. Therefore, the levels of edema regression and prognostic effects of anti-VEGF treatments differ, making an accurate prognosis nearly impossible. Therefore, this retrospective study aimed to explore the impact of anti-VEGF treatment on the thickness of each layer of the retina in patients with ME secondary to BRVO.

## Methods

### Study design and population

This retrospective study included patients with ME secondary to BRVO diagnosed in Ningxia Eye Hospital from January to December 2020. The inclusion criteria were [1] received anti-VEGF therapy (Ranibizumab) and [2] available optical coherence tomography (OCT) data. The exclusion criteria were [1] follow-up duration < 3 months, [2] retinal detachment, glaucoma, optic nerve disease, uveitis, and retinal artery occlusion, [3] history of retinal laser photocoagulation, intravitreal injection, or ocular vitreous surgery, [4] history of the local or systemic use of corticosteroids in the past year, [5] incomplete data, or [6] inability of OCT to determine the morphology of ME.

The patients showing > 25% reduction in the central retinal thickness (CRT) after anti-VEGF therapy were defined as in the response group, while those showing a ≤25% reduction in CRT were included in the no-response group.

The study was approved by the People’s Hospital of Ningxia Hui Autonomous Region committee and followed the tenets of the Declaration of Helsinki. Due to the retrospective nature of the study, the requirement for informed consent from the patient was waived by the approval committee.

### Data collection and definition

**Fig. 1:**
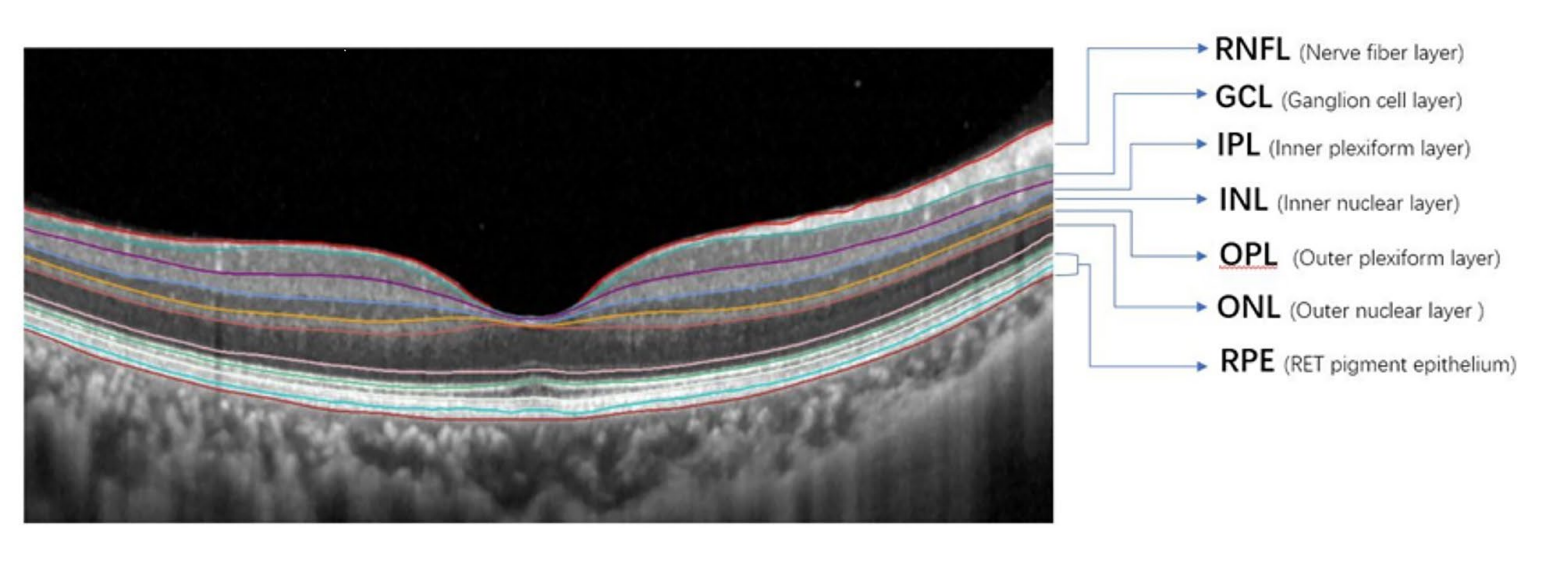
Representation of the automatic segmentation of the retinal layers provided by the Heidelberg software segmentation tool in a foveal B-scan

The data were extracted from the hospital’s medical records. The clinical characteristics of the patients included age, sex, disease course (i.e., time from loss of vision to diagnosis), diabetes mellitus, and hypertension. The best-corrected visual acuity (BCVA), macular fovea or CRT, and retinal thickness, including retinal nerve fiber layer (RNFL), ganglion cell layer (GCL), IPL, INL, OPL, ONL, and retinal pigment epithelium (RPE) (Fig. 1) were collected from the patient charts before treatment and 1, 2, and 3 months after starting the treatment. Mainly influenced by the “*Guidelines for the Management of Retinal Vein Occlusion*” proposed by the European Society of Retina Specialists (EURETINA) and combined with the local medical insurance policy, the 3 + *pro re nata* (3 + PRN) scheme was used for treatment in all patients, i.e., injection once a month in the first 3 months and subsequent injection as needed, according to the morphological response classification of age-related macular degeneration proposed by Amoaku et al. [14]. The response group was defined as a > 25% reduction in CRT compared with the baseline thickness one month after treatment.

**Fig. 2:**
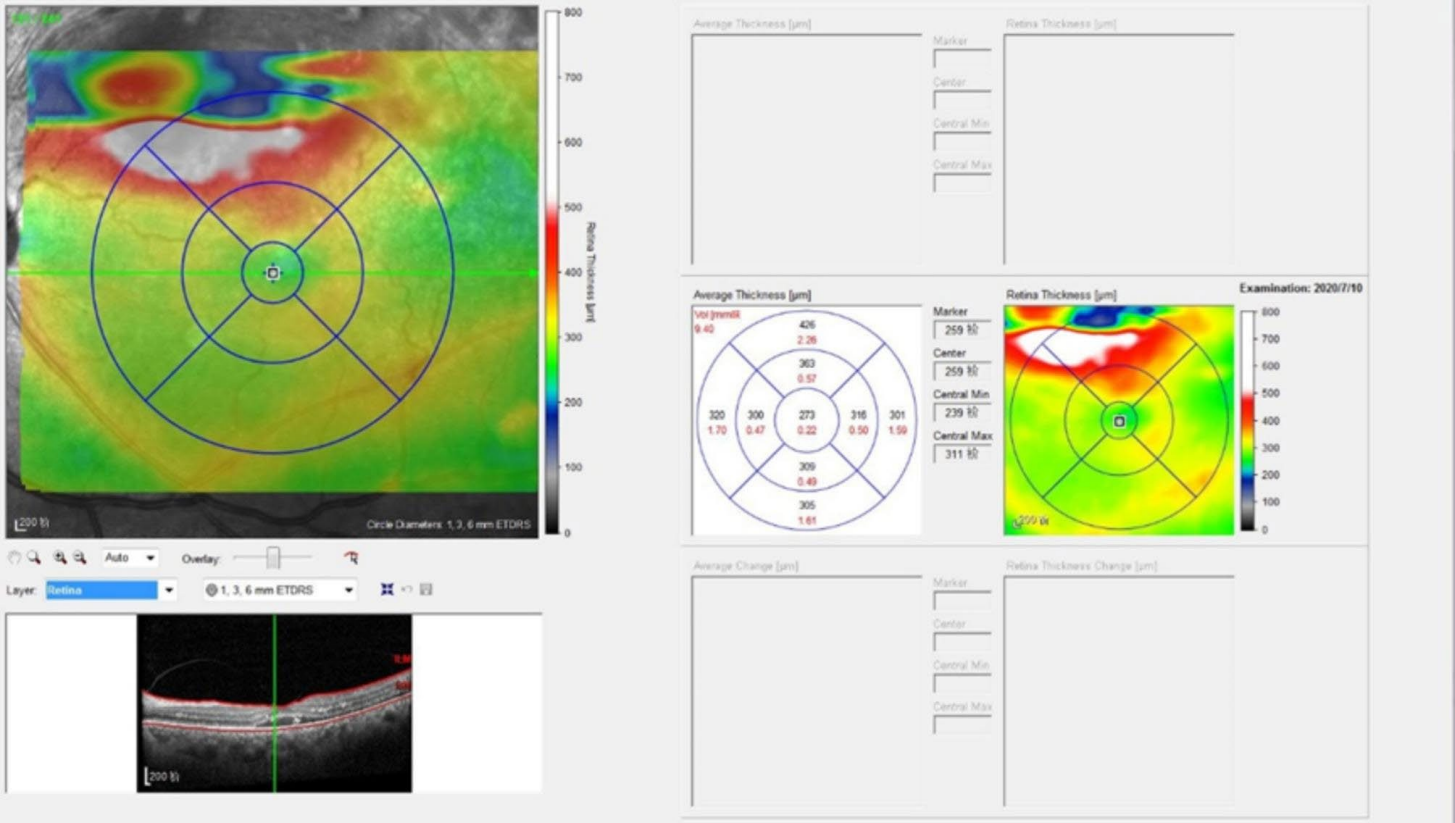
Retinal layers output, with corresponding thickness display according to the ETDRS grid

The BCVA was measured by the International standard visual acuity chart, and the results were converted into the logarithm of the minimum angle of resolution (logMAR), and the logMAR visual acuity was statistically analyzed [15]. A segmentation of all retinal layers was performed for each eye to analyze the retinal thickness as follows: first, automatic segmentation was applied to the complete scan by using the in-built feature for automated segmentation in the Heidelberg Eye Explorer software (Version 1.10.4.0). After that, a manual correction was performed using the appropriate software tools whenever necessary. Both the automatic segmentation and the necessary manual corrections were conducted by a trained grader. Afterward, the eye tracking mode was chosen for obtaining the follow-up images, and images of the exact lesion location at different follow-up times were obtained. According to the location of the lesion, the retinal thickness was determined in the inner circles (3 mm diameter, superior or inferior) (Fig. 2).

### Statistical analysis

Data were analyzed using SPSS 19.0 (IBM, Armonk, NY, USA). BCVA was converted into LogMAR according to the Holladay method. Data were presented as means ± standard deviations (SD) for continuous variables and n (%) for categorical variables. For continuous variables, the difference in the mean changes from baseline between the two groups was evaluated using Student’s *t*-test. A generalized estimating equation (GEE) model was used to estimate the difference in the thickness of each layer of the retina at each time point between the two groups and the time trend after treatment. Two-sided P-values < 0.05 were considered statistically significant.

## Results

Forty-three patients (25 males) were included, 31 of whom showed > 25% reduction in central retinal thickness (CRT) after anti-VEGF therapy (response group), and the others showed a ≤25% reduction in CRT (no-response group). There were significant differences in sex and disease course between the response and no-response groups (all P < 0.05) (Table 1).

**Table 1:** Characteristics of patients between response group and no-response group. Data is shown as n (%) or mean ± standard deviation

Characteristics	Response group (n = 31)	No response group (n = 12)	P
Sex (male/female)	18 (58.1)/13 (41.9)	7 (58.3)/5 (41.7)	< 0.001
Age (years)	57.1 ± 8.6	59.4 ± 10.1	0.250
Course of disease (days)	32.3 ± 10.6	38.3 ± 12.6	< 0.001
Diabetes mellitus	17 (54.8)	6 (50.0)	0.210
Hypertension	14 (45.2)	7 (58.3)	0.450

The INL (at 3 months, P = 0.001), OPL (at 2 and 3 months, both P < 0.001), ONL (at 1, 2, and 3 months, P = 0.007, P < 0.001, P < 0.001, respectively), and CRT (at 1, 2, and 3 months, P < 0.001, P < 0.001, P = 0.040, respectively) were relatively thinner in the response group than in the non-response group (Table 2). Moreover, the results of the mean percentage changes (95% confidence interval) from their corresponding baseline values for the thickness of each retina layer showed that the differences in GCL (at 2 months), IPL (at 1, 2, and 3 months), INL (at 2 and 3 months), OPL (at 3 months), and CRT (at 1, and 2 months) were significantly different between the two groups (all P < 0.05). The GEE model showed significant differences in the mean changes in the thickness of each layer of the retina at GCL (P = 0.045) and IPL (P = 0.006) between the two groups after controlling for the time effect. Furthermore, a significant time trend of the thickness loss was observed, as assessed by the GEE model following repeated measurement of the thickness of each layer of the retina (P < 0.001), except for the GCL (P = 0.086) and RPE (P = 0.644) (Table 3). Additionally, patients in the response group were more likely to have an improvement in IPL (43.68 ± 6.01 at 1 month, 41.52 ± 5.45 at 2 months vs. 39.9 ± 6.86 at baseline) after anti-VEGF therapy, while those in no response group might show improvement in GCL (45.75 ± 8.24 at 1 month, 40.00 ± 8.92 at 2 months, and 38.83 ± 9.93 at 3 months vs. 49.67 ± 6.83 at baseline) (Table 2).

**Table 2:** Thickness of various retinal layers after anti-VEGF treatment for 1, 2, and 3 months in the response group and no-response group. Data is shown as mean ± standard deviation and difference is 95% co-efficient index

Variables	Response group (n = 31)	No-response group (n = 12)	Difference (95% CI)	P
Nerve fiber layer				
Baseline	136.35 ± 27.5	147.08 ± 19.14	(28.25, 6.80)	0.223
1 month	107.06 ± 28.78	105.5 ± 19.85	(-16.76, 19.88)	0.864
2 months	51.35 ± 20.28	52.5 ± 19.87	(-15.00, 12.71)	0.868
3 months	51.35 ± 20.28	30.92 ± 8.26	(-5.45, 7.16)	0.785
Ganglion cell layer				
Baseline	44.13 ± 6.59	49.67 ± 6.83	(-10.11, -0.97)	0.019
1 month	44.23 ± 6.38	45.75 ± 8.24	(-6.28, 3.23)	0.521
2 months	41.81 ± 8.69	40.00 ± 8.92	(-4.20, 7.82)	0.547
3 months	41.26 ± 12.27	38.83 ± 9.93	(-5.60, 10.45)	0.545
Inner plexiform layer				
Baseline	39.9 ± 6.86	50.17 ± 4.73	(-14.63, -5.90)	< 0.001
1 month	43.68 ± 6.01	44.67 ± 6.36	(-5.18, 3.20)	0.636
2 months	41.52 ± 5.45	43.42 ± 4.89	(-5.54, 1.74)	0.298
3 months	38.52 ± 4.78	39.75 ± 4.77	(-4.51, 2.05)	0.452
Inner nuclear layer				
Baseline	58.13 ± 5.54	53.33 ± 3.47	(1.32, 8.27)	0.008
1 month	46.23 ± 7.06	46.00 ± 5.2	(-4.31, 4.76)	0.920
2 months	49.32 ± 7.78	50.42 ± 7.28	(-6.35, 4.16)	0.676
3 months	40.58 ± 6.64	48.92 ± 6.27	(-12.83, -3.84)	0.001
Outer plexiform layer				
Baseline	35.87 ± 2.33	36.42 ± 2.5	(-2.18, 1.09)	0.504
1 month	34.45 ± 5.81	36.08 ± 6.5	(-5.75, 2.49)	0.288
2 months	33.94 ± 4.72	35.75 ± 5.55	(-11.22, -7.53)	< 0.001
3 months	27.29 ± 2.71	36.67 ± 2.61	(-11.22, -7.53)	< 0.001
Outer nuclear layer				
Baseline	114.97 ± 40.88	128.75 ± 18.93	(-38.72, 11.15)	0.271
1 month	89.77 ± 28.99	114.08 ± 6.17	(-41.48, -7.14)	0.007
2 months	75.81 ± 19.5	112 ± 25.34	(-50.76, -21.62)	< 0.001
3 months	69.52 ± 11.32	114.25 ± 17.13	(-53.75, -35.72)	< 0.001
Pigment epithelium				
Baseline	17.1 ± 2.09	17.42 ± 2.15	(-1.77, 1.13)	0.657
1 month	17.55 ± 3.37	18.25 ± 3.39	(-3.02, 1.62)	0.545
2 months	17.90 ± 2.71	18.5 ± 2.58	(-2.43, 1.24)	0.516
3 months	17.94 ± 3.44	18.75 ± 3.36	(-3.16, 1.54)	0.488
Central retinal thickness				
Baseline	505.48 ± 27.28	512.42 ± 28.33	(-25.86, 11.99)	0.464
1 month	322.48 ± 32.76	396.08 ± 33.2	(-96.17, -51.02)	< 0.001
2 months	276.74 ± 23.2	321 ± 32.96	(-62.23, -26.28)	< 0.001
3 months	269.97 ± 20.83	285.58 ± 23.66	(-30.46, -0.77)	0.040
BCVA				
Baseline	0.21 ± 0.13	0.33 ± 0.22	(-0.05, 0.11)	0.436
1 month	0.33 ± 0.22	0.27 ± 0.13	(-0.08, 0.19)	0.412
2 months	0.5 ± 0.33	0.42 ± 0.21	(-0.13,0.29)	0.437
3 months	0.52 ± 0.33	0.45 ± 0.21	(-0.13, 0.28)	0.477

CI: confidence interval; BCVA: best-corrected visual acuity

**Table 3:** Mean changes (SD) in the thickness of different retinal layers from baseline in the response group and the no-response group after treatment for 1, 2, and 3 months

Indicator	Response group (n = 31)	No-response group (n = 12)	Difference of mean changes (95% CI)	P ^a^	P ^b^	P for time trend ^c^
Retinal nerve fiber layer (RNFL)						
1 month	-29.29 ± 3.10	-41.58 ± 19.87	(-0.06, 24.64)	0.051	0.051	< 0.001
2 months	-85 ± 26.33	-94.58 ± 22.31	(-7.80, 26.97)	0.272		
3 months	-104.58 ± 27.31	-116.17 ± 19.86	(-5.94, 29.11)	0.189		
Ganglion cell layer (GCL)						
1 month	0.1 ± 5.06	-3.92 ± 8.33	(-0.18, 8.21)	0.060	0.045	0.086
2 months	-2.32 ± 8.59	-9.67 ± 8.26	(1.51, 13.18)	0.015		
3 months	-2.87 ± 13.5	-10.83 ± 9.39	(-0.64, 16.57)	0.069		
Inner plexiform layer (IPL)						
1 month	3.77 ± 5.07	-5.5 ± 4.81	(5.84, 12.71)	< 0.001	0.006	< 0.001
2 months	1.61 ± 5.19	-6.75 ± 5.24	(4.79, 11.93)	< 0.001		
3 months	-1.39 ± 4.16	-10.42 ± 5.7	(5.86, 12.20)	< 0.001		
Inner nuclear layer (INL)						
1 month	-11.9 ± 8.44	-7.33 ± 5.88	(-9.95, 0.81)	0.094	0.193	< 0.001
2 months	-8.81 ± 7.43	-2.92 ± 6.58	(-10.84, -0.936)	0.021		
3 months	-17.55 ± 7.52	-4.42 ± 6.29	(-18.08, -8.18)	< 0.001		
Outer plexiform layer (OPL)						
1 month	-1.42 ± 6.2	-0.33 ± 6.75	(-5.447, 3.275)	0.618	0.149	< 0.001
2 months	-1.94 ± 4.33	-0.67 ± 4.7	(-4.310, 1.773)	0.404		
3 months	-8.58 ± 2.96	0.25 ± 1.71	(-10.68, -6.99)	< 0.001		
Outer nuclear layer (ONL)						
1 month	-25.19 ± 19.69	-14.67 ± 17.2	(-23.61, 2.56)	0.112	0.160	< 0.001
2 months	-39.16 ± 25.1	-16.75 ± 28.23	(-40.25, -4.57)	0.015		
3 months	-45.45 ± 31.62	-14.5 ± 19.81	(-50.81, -11.09)	0.003		
Retinal pigment epithelium (RPE)						
1 month	0.45 ± 2.45	0.83 ± 2.44	(-2.06, 1.30)	0.649	0.570	0.644
2 months	0.81 ± 1.83	1.08 ± 1.68	(-1.51, 0.95)	0.652		
3 months	0.84 ± 2.92	1.33 ± 2.87	(-2.49, 1.50)	0.620		
Central retinal thickness (CRT)						
1 month	-183 ± 24.33	-116.33 ± 22.79	(-83.09, -50.24)	< 0.001	0.253	< 0.001
2 months	-228.74 ± 31.31	-191.42 ± 24.96	(-57.75, -16.90)	0.001		
3 months	-235.52 ± 28.03	-226.83 ± 25.61	(-27.50, 10.13)	0.357		
Best-corrected visual acuity (BCVA)						
1 month	0.12 ± 0.13	0.09 ± 0.1	(-0.06, 0.11)	0.552	0.659	< 0.001
2 months	0.29 ± 0.25	0.24 ± 0.17	(-0.11, 0.21)	0.537		
3 months	0.31 ± 0.25	0.27 ± 0.18	(-0.12, 0.20)	0.595		

^a^ Comparison of the mean changes from the respective baseline between the response and no-response groups at 1, 2, and 3 months after treatment; two-sample t-test

^b^ Comparison of the mean changes from the respective baseline between the response and no-response groups using the GEE methods to control the time effect in the repeated measurement

^c^ Repeated measurement of the time trend in the GEE model

CI: confidence interval; GEE: generalized estimating equation

## Discussion

This retrospective study suggests that anti-VEGF treatment might help improve the retinal structure and function over the first 3 months in patients with ME secondary to BRVO, and those who have a response after anti-VEGF therapy could be more likely to have an improvement in IPL, while those having no response might show improvement in GCL. Overall, although the clinical applicability is limited, the findings of this study could help improve the understanding of BRVO and anti-VEGF treatment.

In this study, the RNFL values in both groups decreased after anti-VEGF treatment, but there were no significant differences between the two groups after 3 months of treatment. Studies have shown that BRVO decreases the RNFL thickness, but this decrease could also be due to a variety of factors besides RVO, including glaucoma and various systemic diseases such as diabetes, hypertension, and carotid artery ischemia [16–18]. The exact contribution and interrelationships of these factors remain to be quantified.

The GCL consists of the nucleus of the ganglion cells and third-order neurons in the retina. VEGF protects the retinal ganglion cells and can promote tissue development and maturation [19]. The anti-VEGF treatment of BRVO-secondary ME accelerates ganglion cell death, and the GCL thickness will become thinner and even atrophied after repeated anti-VEGF injections [19]. In this study, the GCL value of the no-response group decreased at 1, 2, and 3 months after treatment compared with baseline (Table 2). At the last follow-up, the values were lower than the standard range of the GCL (68–101 μm [20]). Therefore, anti-VEGF therapy appears to affect the GCL in some patients with a poor response. It also indicates that, after a long period of treatment, the GCL thickness in these patients might be lower than normal or even become atrophied.

The IPL is a loose connective tissue formed by the contact of bipolar cells, non-elongated cells, and ganglion cells to form synapses. Anti-VEGF drugs subside the retinal IPL layer edema in BRVO-secondary ME; even after anti-VEGF treatment, the IPL gradually becomes thinner [18, 21]. In this study, the IPL thickness of the two groups of patients after treatment was reduced compared with before treatment (P < 0.05). Although within the normal value (28–88 μm) [22], literatures suggesting that atrophy and thinning may occur after treatment [18, 21]. Therefore, the anti-VEGF treatment led to subsided IPL edema, and the thickness after treatment was reduced compared with before.

The results suggested that the INL thickness decreased in both groups after anti-VEGF treatment, but the response group showed a decrease at 2 months of treatment. VEGF plays a vital role in the development of ME and BRVO. Persistently high VEGF in patients with BRVO leads to vascular leakage and ME [18, 23, 24]. Therefore, the VEGF levels were decreased after anti-VEGF treatment, contributing to decreasing the INL thickness. Despite the same treatment, the patients in the response group showed some improvement in the structural disorganization of the INL after anti-VEGF therapy. Nevertheless, the damage to the INL in this part was only mild. It could suggest an incomplete destruction of the inner retinal cells since they showed a bipolar, horizontal, and non-elongated morphology. Thus, this damage was possibly insufficient to block the transmission of information from the photoreceptors to the ganglion cells [25, 26]. These results support the changes in the inner retinal layers after anti-VEGF treatment [27]. Still, additional studies are necessary for confirmation.

The OPL is a synaptic site with rod cells connected with the dendrites of bipolar cells, the processes of the horizontal cells, and loose connective tissue. When patients develop BRVO, increased venous hydrostatic circulation pressure and abnormal perfusion in the outer retina can lead to increased VEGF leakage from adjacent vessels. Due to the lack of a vascular system in the outer retina, fluid clearance from the central retina is reduced, and a large amount of fluid accumulates in the OPL. When ME occurs, the thickness of the OPL increases [28, 29]. Studies showed that the degree of edema in the OPL and ONL at baseline represents the degree of photoreceptor damage. It also indicates that the visual acuity and anatomical recovery effects after anti-VEGF treatment can be related to OPL and ONL [30]. Nevertheless, the changes in the OPL thickness after anti-VEGF therapy in BRVO patients have not been clearly proposed. In this study, only the response group showed a statistically significant decrease in OPL thickness after treatment compared with baseline (P < 0.05), and a significant difference was found in the thickness of the OPL at 3 months after treatment compared with the non-response group. Therefore, the effect of OPL edema subsiding might possibly be more obvious after at least three regular treatments. It will have to be confirmed.

The ONL is the thickest layer in the fovea retina, mainly composed of photoreceptor nuclei and occupying the highest proportion of the retina. Changes in ONL BRVO directly affect the overall retinal thickness. In this study, after three regular anti-VEGF treatments, the ONL thickness of the patients in the response group was significantly reduced compared with the baseline (P < 0.05). In the comparison between the two groups, in the first, second, and third months after treatment, the response group showed a regression of external nuclear layer edema compared with the non-response group. Only a few studies reported that the ONL is related to visual acuity and retinal function. Ota et al. [31] believed that when patients developed BRVO secondary to ME, retinal ischemia and hypoxia would persist, which would eventually lead to different degrees of atrophy and thinning of the inner five layers of the retinal artery supply, but the changes in visual function were not correlated with the inner five layers. Altunel et al. [32] found in a follow-up study on the changes of photoreceptors in patients with BRVO that the thickness of the ONL was correlated with visual acuity and believed that the thickness of the ONL could effectively evaluate the integrity of the outer membrane in patients. Still, no significant associations were found in the present study. Therefore, after applying anti-VEGF therapy, the ONL thickness decreased in both response and no-response groups, while response group decreased more obvious.

In this study, the thickness of pigment epithelium did not change after anti-VEGF treatment. The pigment epithelium contains a large amount of melanin, constituting the retina’s outer barrier, and the cells are closely connected. Usually, the pigment epithelium layer is not prone to edema in ME and should not be affected by the anti-VEGF agents [33]. The results showed no significant difference in BCVA between the response and the no-response groups after 3 months of anti-VEGF treatment, which might be mainly related to the short follow-up time of the two groups. It suggests that intravitreal injection of VEGF could improve the visual function, as supported by previous studies [34, 35], but without returning to normal values, at least within 3 months. Amoaku et al. [14]suggested that there would be significant differences in treatment outcomes after 12 months of treatment. In cases of poor response to anti-VEGF therapy, such patients should be re-evaluated and, if necessary, replaced with alternative therapies, including other types of anti-VEGF agents, based on the characteristics of the lesion. In some cases, treatment can be permanently discontinued if it is deemed that further treatment will not be beneficial, i.e., due to the lesion morphology, including retinal scarring. Therefore, grouping patients over a short period may allow rapid assessment of the best treatment, and clinicians may consider changing treatment to other agents for patients in the non-responsive group.

It has been reported that BRVO mostly occurs in the superior temporal quadrant, followed by the inferior temporal quadrant [36, 37]. It could be because the retinal range supported by the blood vessels on the temporal side is larger than on the nasal side, and the blood vessels have a longer course and more branches. In addition, the nasal veins are often smaller or even absent. In addition, blood perfusion has been reported to be higher in the superior temporal branch [36, 37]. People often stand in their daily activities. When the velocity and volume of blood flow are reduced due to the standing position and gravity, venous obstruction can occur. Harder arteries due to arteriosclerosis can also lead to blood stasis and obstruction [36, 37].

In addition, the available epidemiological data suggest that the incidence of branch retinal vein occlusion is more common in males than in females, with a male/female ratio of 1.3:1-1.8:l, which might be due to the differences in sex hormones [38]. We believe that sex hormones, especially estrogen, might be related to this disease through their effects on vascular structure and blood viscosity. The present study also showed that BRVO was more common in men than women (25 males vs. 18 females). However, there is no statistical significance, which may be caused by the small sample size.

Despite the promising results and important findings, this study has limitations. First, this study was performed at a single center, and the sample size was small, with only 43 patients who were screened from 200 patients by the inclusion and exclusion criteria. Second, because of using the 3 + PRN treatment approach, the treatment effects after 3 months were much different among the patients and, thus, were not included, which limited the study of evaluating the effects of anti-VEGF only to 3 months. Third, the adverse events to anti-VEGF treatment were not analyzed because of inconsistent reporting in the available charts. Fourth, this study only examined the quantifiable retinal layers, and the impact of the integrity of the external membrane and PL on retinal layers was not studied.

## Conclusions

Anti-VEGF therapy might help restore retinal structure and function in patients with ME secondary to BRVO, and those who have a response after anti-VEGF therapy might be more likely to show IPL improvements, while those having no response might show improvement in the GCL. The findings also suggest that anti-VEGF treatment could counter the effect of VEGF and subsequent occurrence of ME secondary to RVO and could be a useful approach for restoring the retinal structure and function over the first 3 months in treating ME secondary to BRVO. Further investigation on the effect of anti-VEGF treatment in treating ME secondary to BRVO for an extended period is warranted. Likewise, an investigation is recommended for studying the localization of vein occlusion, especially in branch occlusion, as upper BRVO develops ME more frequently than the lower branch occlusion.

## Data Availability

All data generated or analysed during this study are included in this published article.

## List of Abbreviations

RVO: Retinal vein occlusion
BRVO: Branch retinal vein occlusion
CRVO: Central retinal vein occlusion
ME: Macular edema
VEGF: Vascular endothelial growth factor
OCT: Optical coherence tomography
FFA: Fundus fluorescein angiography
CRT: Central retinal thickness
BCVA: Best corrected visual acuity
ANOVA: Analysis of variance
Log MAR: Logarithm of the minimum angle
RNFL: Retinal nerve fiber layer
GCL: Ganglion cell layer
IPL: Inner plexiform layer
INL: Inner nuclear layer
OPL: Outer plexiform layer
ONL: Outer nuclear layer
PL: Photoreceptor layer
RPE: Retinal pigment epithelium
IS/OS: Photoreceptor inner segment/photoreceptor outer segment
